# Delayed Presentation of Post-traumatic Coronary Artery Dissection Resulting in Acute Myocardial Infarction and Severe Left Ventricular Dysfunction Treated With Angioplasty and Stenting: A Case Report

**DOI:** 10.7759/cureus.94069

**Published:** 2025-10-07

**Authors:** Ashish Gupta, Nitish Garg

**Affiliations:** 1 Internal Medicine, Cardionova Institute of Medical Sciences, Jalandhar, IND; 2 Cardiology, Cardionova Institute of Medical Sciences, Jalandhar, IND

**Keywords:** acute myocardial infarct, angioplasty and stenting, left anterior descending artery (lad), post-traumatic coronary artery dissection, traumatic coronary artery dissection

## Abstract

This case report describes a 32-year-old male patient who, two months after sustaining blunt chest trauma in a motor vehicle accident, presented with acute myocardial infarction (MI). Upon initial trauma evaluation abroad, it was reported that he had an unremarkable electrocardiogram (ECG) and chest CT and had remained asymptomatic. Two months later, he developed exertional chest pain and shortness of breath, prompting emergent reevaluation. The ECG demonstrated anterolateral ST-segment elevations, and cardiac troponins were markedly elevated (30-40 ng/L). Coronary angiography confirmed a dissection of the proximal left anterior descending (LAD) artery. The patient underwent successful angioplasty with stent placement. Despite successful revascularization, he developed severe heart failure with reduced ejection fraction (HFrEF), requiring guideline-directed medical therapy (GDMT), which showed improved ejection fraction (EF) from 27% to 30%-35% on follow-up. This case represents a delayed presentation of post-traumatic coronary artery dissection, given the temporal association with prior blunt chest trauma, absence of traditional atherosclerotic risk factors, and angiographic findings. It underscores the importance of maintaining high clinical suspicion for coronary injury in patients with a history of chest trauma, even after an initially normal evaluation.

## Introduction

Post-traumatic coronary artery dissection is an uncommon but potentially life-threatening complication of blunt chest trauma, capable of leading to myocardial infarction (MI), sudden cardiac death, or severe cardiac dysfunction if undetected [1,2,3]. Most published data are limited to case reports, underscoring its rarity, with an overall incidence estimated at 0.1%, coronary artery dissection represents the least common form of such injury. [4] The possibility of delayed symptom onset poses significant diagnostic challenges. The pathophysiologic mechanisms behind blunt cardiac injury include direct compression of the heart between the sternum and spine, rapid deceleration causing shearing forces on the coronary arteries at their fixation points, a sudden increase in intracardiac pressure leading to intimal tears, and penetration by displaced rib fractures. [5] The left anterior descending (LAD) artery is typically the most affected vessel, and percutaneous coronary intervention (PCI) is the recommended therapeutic strategy [6,7]. While some patients present acutely, delayed presentations are well-documented, with symptoms typically developing hours to days post-injury. [3] Maintaining a high index of suspicion for coronary artery dissection in patients presenting with chest symptoms post-trauma, regardless of symptom-free intervals, is crucial for timely intervention. This case is distinctive for its unusually long two-month asymptomatic interval, extending well beyond previously reported timelines and underscoring the diagnostic complexity of trauma-associated coronary injury.

## Case presentation

A 32-year-old male patient with no significant medical history experienced a high-impact vehicular accident while traveling abroad. The initial evaluation at a local hospital abroad, which, according to the patient's report, included a CT scan of the chest and an electrocardiogram (ECG) performed, was unremarkable, and he remained asymptomatic following discharge. Two months later, while visiting family, the patient developed acute-onset exertional chest pain and progressive shortness of breath, prompting him to seek reevaluation at our emergency department.

Diagnostic assessment

**Figure 1:**
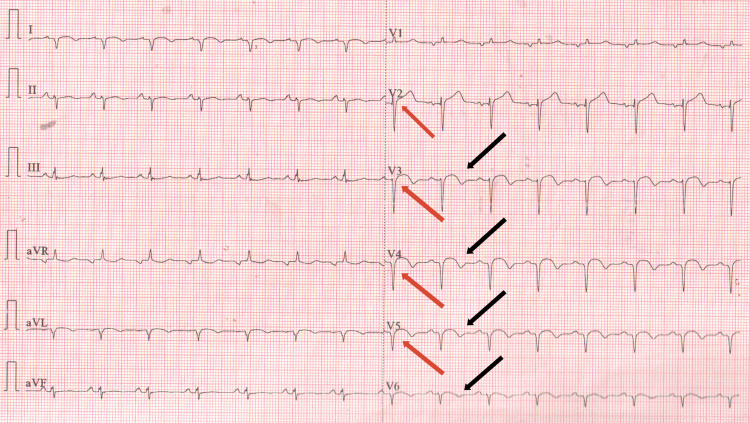
ECG report ST elevations (red arrows) and T wave inversions (black arrows) show anterolateral wall myocardial infarction.

**Figure 2:**
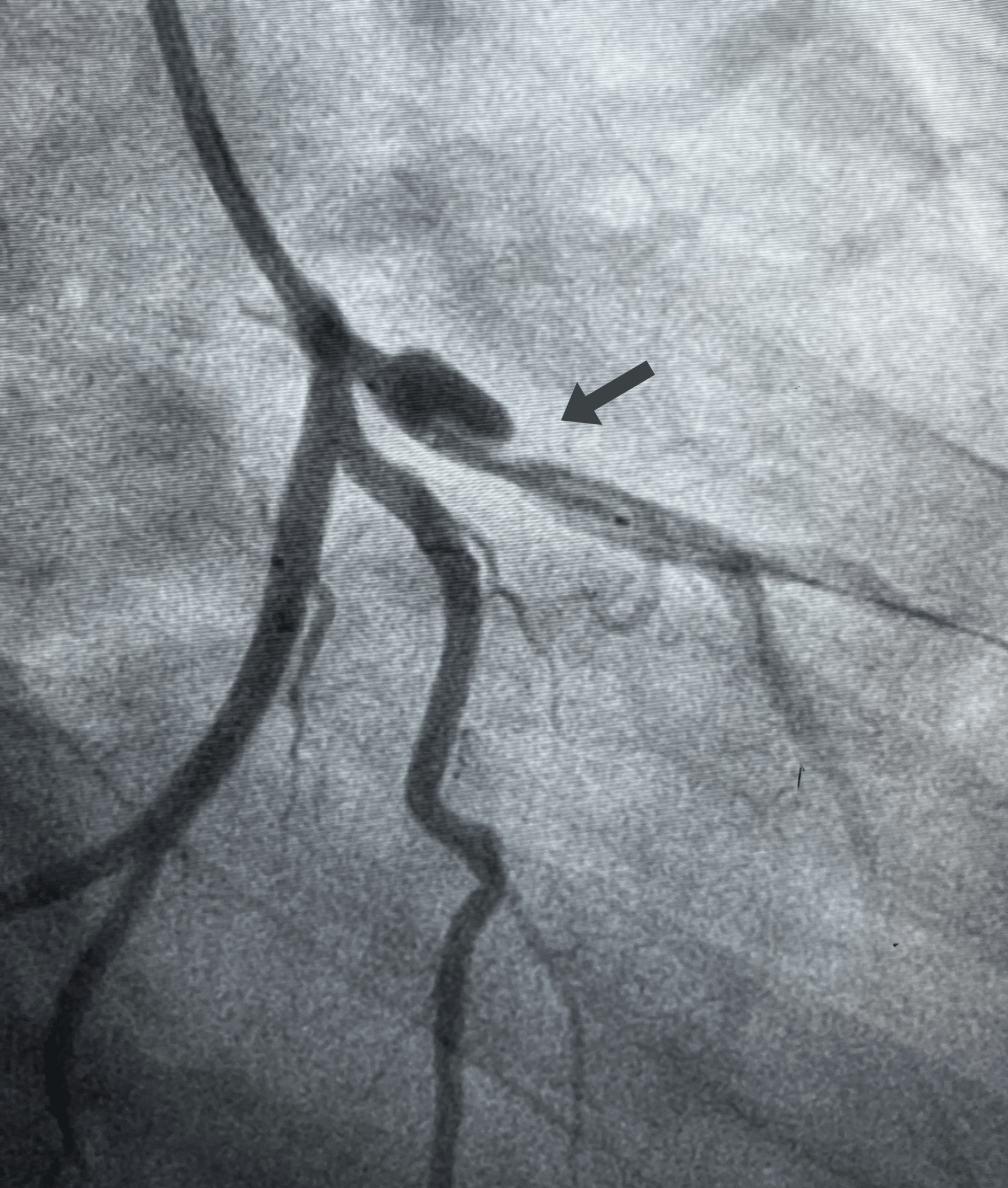
Coronary angiography Stenosis and dissection of proximal left anterior descending (LAD) artery.

Upon reevaluation, a repeat ECG demonstrated new ST elevations and T wave inversions indicative of anterolateral myocardial infarction (Figure 1). Elevated high-sensitivity cardiac troponins were consistent with acute MI. Serial troponin measurements were not pursued further once the diagnosis was established and emergent management was initiated. Transthoracic echocardiography revealed severe left ventricular systolic dysfunction with an ejection fraction (EF) of 27% and anterior and septal wall akinesis. Coronary angiography identified stenosis and a dissection in the proximal left anterior descending (LAD) artery (Figure 2), confirming the diagnosis of coronary artery dissection, which-given the patient’s prior chest trauma, absence of risk factors, and clinical context-was most consistent with a post-traumatic etiology.

Therapeutic intervention

**Figure 3:**
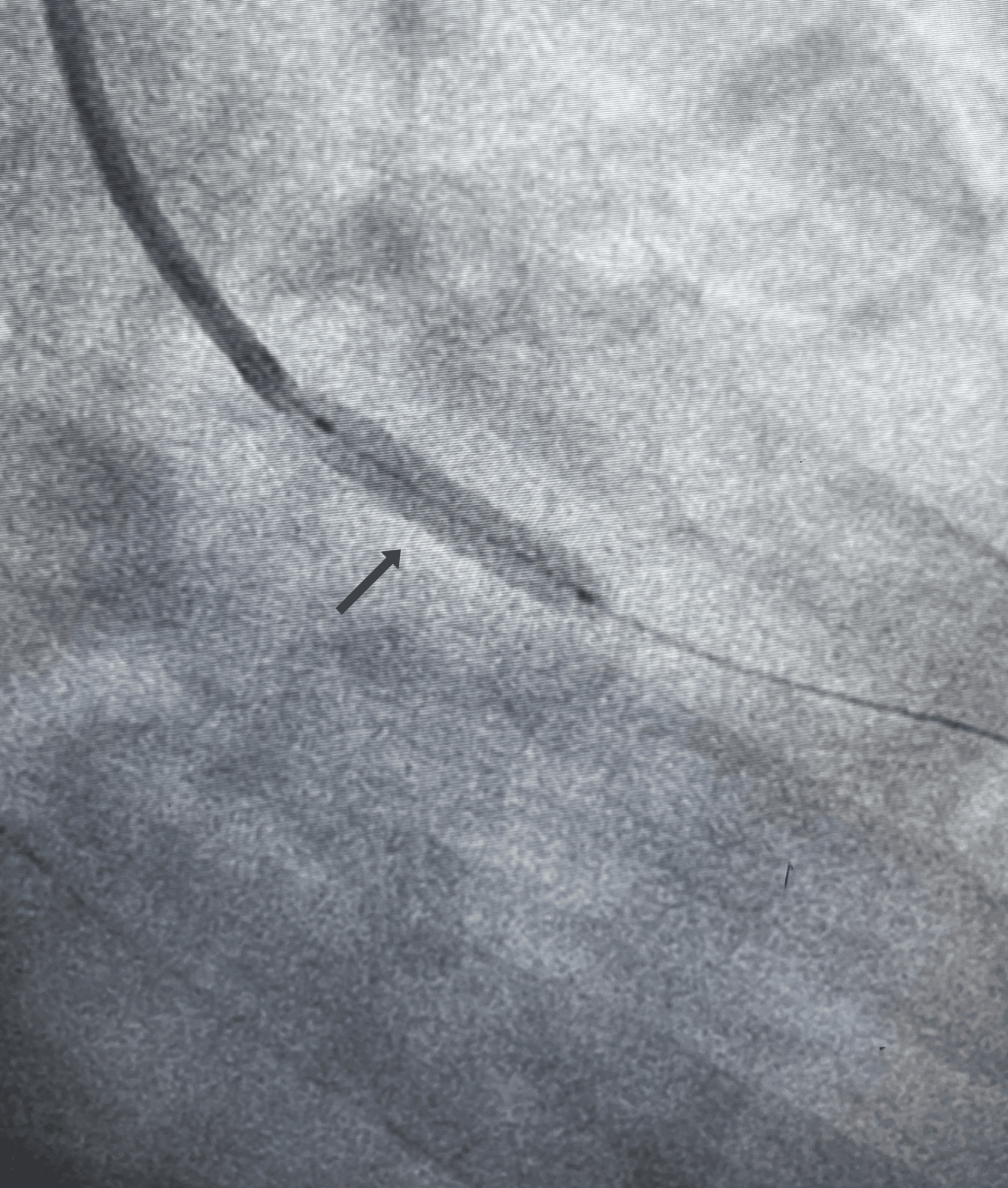
Coronary angioplasty Stent deployed in left anterior descending (LAD) artery.

**Figure 4:**
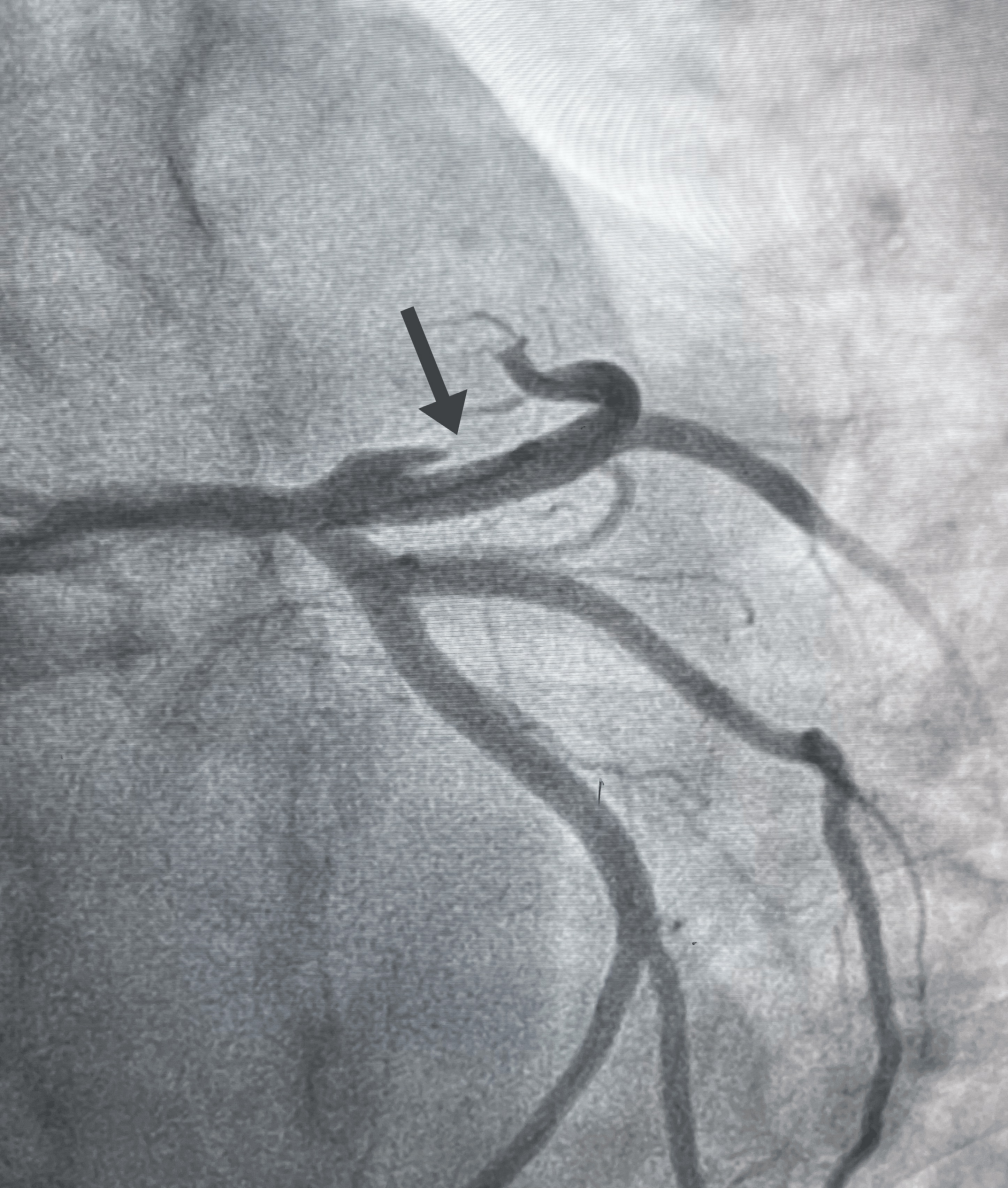
Coronary angioplasty Deployed left anterior descending (LAD) artery stent with restored blood flow, correcting stenosis and dissection.

The patient underwent emergent percutaneous coronary intervention (PCI) with angioplasty and drug-eluting stent deployment in the proximal LAD artery. (Figures 3, 4), successfully restoring coronary flow and resolving the dissection. Post-procedure, guideline-directed medical therapy (GDMT) was initiated to manage heart failure with reduced ejection fraction (HFrEF). [8] His regimen included dual antiplatelet therapy and a comprehensive heart failure cocktail consisting of sacubitril/valsartan, spironolactone, and ivabradine. Once the acute phase had resolved and he was hemodynamically stable, a beta-blocker was carefully added to his regimen.

Follow-up and outcome

Post intervention, the patient was closely monitored and exhibited symptomatic improvement consistent with New York Heart Association (NYHA) Class I functional status. A follow-up echocardiogram at two months post-intervention showed an improvement in EF of 30%-35% with persistent left ventricular dysfunction. He was enrolled in a cardiac rehabilitation program and received extensive counseling on diet and exercise.

## Discussion

This case emphasizes the rare occurrence of unusually delayed symptomatic presentation following post-traumatic coronary artery dissection. The initial asymptomatic course and normal ECG findings, combined with a two-month latent period, significantly exceed the more commonly reported timelines of hours to days, highlighting the need for a high index of suspicion to be maintained long after the initial traumatic event and underlining the necessity for clinicians to remain vigilant. Post-traumatic coronary artery dissection should be included in the differential diagnosis of patients presenting with chest symptoms or unexplained cardiac dysfunction, even months after a chest injury, given the potential for delayed onset of significant symptoms [3,4,9].

The anatomical susceptibility of the LAD artery due to its anterior chest positioning increases vulnerability during blunt trauma [9]. Coronary angiography remains essential for confirming diagnosis, while PCI offers a practical and lifesaving therapeutic option [10]. Although long-term prognosis is not well characterized due to rarity, published case reports suggest that timely intervention can be lifesaving. Conversely, a delayed diagnosis, as seen in this patient, can result in irreversible myocardial injury and severe complications like cardiogenic shock or chronic heart failure. [2,9] The patient's persistent HFrEF, despite successful revascularization, is a direct consequence of the prolonged ischemic time.

While coronary angiography is the diagnostic gold standard, intravascular imaging modalities, such as intravascular ultrasound (IVUS), are highly relevant in these situations. IVUS during PCI can assist in accurate stent placement, reducing complications and improving outcomes [10]. Though not utilized in this case, its application is a key consideration for improving procedural success in future cases.

Long-term management is essential for optimizing outcomes. Implementing comprehensive GDMT, including beta-blockers, angiotensin-converting enzyme (ACE) inhibitors or angiotensin receptor-neprilysin inhibitors (ARNIs), and mineralocorticoid receptor antagonists, after PCI has been shown to significantly improve survival and ventricular function in patients with trauma-induced heart failure [8]. Continued long-term follow-up is recommended to monitor for adverse cardiac events, assess ventricular recovery, and guide adjustments in heart failure management.

## Conclusions

Delayed symptomatic presentation of post-traumatic coronary artery dissection is uncommon but essential to recognize. Clinicians should maintain a high level of suspicion for this diagnosis in patients with a history of chest trauma who present with new cardiac symptoms, even after a symptom-free interval. Early identification and intervention remain critical for favorable outcomes.

In this case, despite PCI and initiation of GDMT, the patient had persistent left ventricular dysfunction at short-term follow-up. This highlights that delayed recognition may lead to irreversible myocardial damage. While longer-term prognosis and optimal follow-up strategies cannot be determined from this single case, literature suggests that comprehensive management, including GDMT, cardiac rehabilitation, and close outpatient monitoring, is a key consideration.

Overall, this case highlights both the lifesaving role of PCI in traumatic coronary dissection and the potential consequences of delayed diagnosis. Clinicians should remain vigilant in evaluating chest pain after trauma and consider coronary injury in the differential diagnosis, even months after the initial event.
